# Healthcare professionals’ understanding of the legislation governing research involving adults lacking mental capacity in England and Wales: a national survey

**DOI:** 10.1136/medethics-2017-104722

**Published:** 2018-04-25

**Authors:** Victoria Shepherd, Richard Griffith, Mark Sheehan, Fiona Wood, Kerenza Hood

**Affiliations:** 1 Division of Population Medicine, Cardiff University, Cardiff, UK; 2 Centre for Trials Research, Cardiff University, Cardiff, UK; 3 College of Human and Health Sciences, Swansea University, Swansea, UK; 4 Ethox Centre, University of Oxford, Oxford, UK

**Keywords:** capacity, third party consent/incompetents, demographic surveys/attitudes, legal aspects, clinical trials

## Abstract

**Objective:**

To examine health and social care professionals’ understanding of the legislation governing research involving adults lacking mental capacity in England and Wales.

**Methods:**

A cross-sectional online survey was conducted using a series of vignettes. Participants were asked to select the legally authorised decision-maker in each scenario and provide supporting reasons. Responses were compared with existing legal frameworks and analysed according to their level of concordance.

**Results:**

One hundred and twenty-seven professionals participated. Levels of discordance between responses and the legal frameworks were high across all five scenarios (76%–82%). Nearly half of the participants (46%) provided responses that were discordant in all scenarios. Only two participants (2%) provided concordant responses across all five scenarios.

**Discussion:**

Participants demonstrated a lack of knowledge about the legal frameworks, the locus of authority and the legal basis for decision-making. The findings raise concern about the accessibility of research for those who lack capacity, the ability to conduct research involving such groups and the impact on the evidence base for their care.

**Conclusion:**

This is the first study to examine health and social care professionals’ knowledge and understanding of the dual legal frameworks in the UK. Health and social care professionals’ understanding and attitudes towards research involving adults with incapacity may warrant further in-depth exploration. The findings from this survey suggest that greater training and education is required.

## Introduction

Informed consent is a fundamental requirement for the ethical and legal conduct of clinical research; however, obtaining valid consent can be particularly problematic in specific practice contexts, where contextualising consent correctly can be uncertain and open to significant disagreement.^1^ Involving individuals who lack capacity to consent to research is essential in order to improve their care and treatment; therefore, legislative provisions permit alternative arrangements for decision-making for such individuals. Under the dual regulatory framework in England and Wales, the Mental Capacity Act 2005 (MCA) has provisions relating to research involving adults lacking capacity, where a relative or friend who is ‘engaged in caring for the person or is interested in their welfare’ is consulted as their personal consultee.^2^ Clinical trials of a medical product are regulated separately by the Clinical Trials Regulations (CTR),^3^ where a personal legal representative who is suitable ‘by virtue of their relationship’ provides informed consent.^3^ In circumstances where a person does not have a friend or relative to act in a personal capacity, there is provision for a professional involved in their care to act as a professional legal representative^3^ or nominated consultee.^2^ The complexity of this dual legislation risks those involved in conducting research in practice settings where individuals may lack capacity failing to meet the ethical and legal requirements.^4^ In this paper, the term proxy is used to refer to those acting as either a consultee or a legal representative.

There are significant differences between decisions relating to medical treatment of adults lacking capacity, which are based on a determination of the person’s best interests, and those concerning their participation in medical research, and also between different types of research.^4^ Both the MCA and CTR require a decision based on what the person lacking capacity would have wanted, had they the capacity to choose for themselves, as their presumed will. The complexity of the current legal framework, its legislative differences and uncertainty surrounding their interpretation has resulted in confusion both for researchers and Research Ethics Committees,^5 6^ as well as clinicians, relatives and carers involved in decisions about adults lacking capacity participating in research.^7^ These differences increase the burden on those involved in making decisions about research participation and present barriers to conducting research with individuals with cognitive impairments.^8 9^ As a result, the exclusion of these groups from research is widespread, and concerns about their exclusion have been reported.^10 11^ Healthcare professionals and social care practitioners are often involved in the identification, provision of information and recruitment processes for research. Their role often involves that of gatekeeper, through which they can allow or deny access to research participation.^12^ A lack of knowledge and understanding by gatekeepers may act as an additional barrier to conducting research.^12^ There is evidence that healthcare professionals have limited knowledge of the legislation governing the care and treatment of those who lack mental capacity,^13^ and also in research contexts,^14–16^ which may impact on their confidence and competence in enrolling those in their care in research studies.

To our knowledge, no studies have examined UK healthcare and social care professionals’ understanding or knowledge of the legal frameworks. This study aimed to examine health and social care professionals’ understanding of the legislation governing proxy consent for research participation by adults lacking capacity in England and Wales.

## Methods

A cross-sectional online survey was conducted^17^ using a series of vignettes.^18 19^ The participants comprised health and social care professionals whose role involved providing care for patients, service users or research participants without mental capacity. Potential participants were invited through special interest professional groups likely to be involved in research involving adults with cognitive impairments. Social media platforms were also used to share details of the survey.

The objective of this study was to obtain descriptive data; therefore, a formal sample size calculation was not performed. The target sample size of approximately 150 participants was estimated from similar studies of Chief Investigators in the USA^16^ and researchers in ageing in Canada.^15^ Participants were provided with information about the study. Prior to completion participants were required to agree with a statement confirming that they were consenting to take part in the study. Participants were also required to confirm that they were a health or social care professional based in England or Wales, and that their role involved the care of patients/service users/research participants, some of whom may be unable to make decisions for themselves (ie, people without mental capacity).

## Questionnaire

The survey was conducted using an electronic survey instrument (Bristol Online Survey) using both fixed-choice and open-ended questions to explore participants’ knowledge of the enrolment process for adults lacking capacity, their understanding of relevant law and comfort with proxy consent for research through five vignettes (see online supplementary appendix 1) describing five hypothetical situations. Each hypothetical study had varying levels of potential risk, with vignettes 1, 3 and 4 stating that there was no serious risk to participants, and both clinical trials (vignette 2 and 5) and other types of research study were included. Sufficient detail was provided to allow identification of the appropriate proxy decision-maker.

10.1136/medethics-2017-104722.supp1 Supplementary data

Participants were asked to indicate in each case who was legally authorised to decide whether the person should take part in the study or not by ticking all applicable answers from a list of carers and professionals involved with the person. Open text boxes were provided to allow respondents to provide explanations for their answers. Participants’ sociodemographic information was also collected. The survey was conducted between February and March 2017.

## Analysis

Survey data were exported and analysed using IBM Statistical Package for the Social Sciences (SPSS software V.23.0) and qualitative data analysis software (NVivo V.11). Textual response data were coded by themes identified both a priori and those that emerged from the data. Data were analysed to describe any associations between participant characteristics and responses that were concordant (or not) with the regulatory frameworks.

Concordance was assessed using the fixed-choice answers and open-text responses. Responses were considered wholly concordant if the legally authorised decision-maker was selected (or included in the choice if multiple options were selected) and the text explicitly supported the reasoning behind the choice (or did not discredit the choice), and partially concordant if the text indicated an understanding of the legally authorised decision-maker or process. Responses were considered to show discord with the legal frameworks if the legally authorised individual was not selected and the text failed to support the true decision-making process or was directly opposed to it. Responses where the open text contained conflicting statements, or the selected option and text conflicted, were categorised as unclear or mixed. Where participants had selected a ‘don’t know’ option or indicated in the text that they were unsure how to respond, responses were categorised as demonstrating uncertainty.

## Results

Following an internal pilot phase to assess understanding and completion of survey items, 127 participants completed the survey (see table 1). Participants were predominantly female (80%), worked in Wales (56%) and had a nursing background (34%). Medical professionals who provided details about their role included general practitioners (GPs) (n=13) and specialists in palliative medicine (n=2). Nursing professionals included Registered Mental Health nurses (n=9), research nurses or researchers (n=10) and those working in palliative care (n=5). Those identifying as allied health professionals were a diverse group that included midwives, clinical psychologists, paramedics, occupational therapists, speech and language therapists, and physiotherapists. Social care practitioners included care home managers (n=12), social workers (n=6) and those who had a role as an independent advocate or Best Interest Assessor (n=4).

**Table 1:** Participant characteristics

Characteristic	Participants n (%)
Country	
England	56 (44%)
Wales	71 (56%)
Gender	
Male	21 (17%)
Female	102 (80%)
Prefer not to say	4 (3%)
Professional background*	
Medical professional	28 (22%)
Nurse	44 (35%)
Allied health professional	29 (23%)
Social care practitioner	28 (22%)
Length of time in profession	
<12 months	1 (1%)
1–2 years	6 (4%)
2–5 years	4 (3%)
5–8 years	11 (9%)
>8 years	105 (83%)
Involvement in research as part of role	
No	47 (37%)
Yes	80 (63%)
If yes *	
In a minor role (research being carried out where I work)	33 (41%)
Informing patients/service users about research studies	37 (46%)
Recruiting participants for research studies	44 (55%)
As a principal investigator at a research site	18 (22%)
As a chief investigator	11 (14%)
Heard about the survey	
Invited through research/professional network or organisation	89 (70%)
Shared on social media	30 (24%)
Other	8 (6%)

*Participants could select more than one option if more than one applied.

Most participants were experienced professionals, having been in their role for >8 years (83%), and many were involved in research (63%) in a range of research roles but most commonly recruiting participants (55%, 44/80).

Responses indicated a broad spectrum of understanding about the legal frameworks, although responses were predominantly discordant, with low levels of understanding and some degree of uncertainty expressed. The number of options selected by participants as being the legally authorised decision-maker was reasonably consistent across all five vignettes (table 2). For research studies where the MCA applies (vignettes 1, 3 and 4), the family member may act as the personal consultee and provide advice about what the person’s wishes would be, if they had capacity in the matter; however, the decision about whether they take part in the study or not lies with the researcher.^2 20^ Where the research is a clinical trial of a medicinal product (vignettes 2 and 5), it is governed by the CTR.^3^ Under the provisions of the Regulations, the family member described in the vignette may act as personal legal representative and provide legally valid informed consent on the person’s behalf^3^ without any legal process such as a Lasting Power of Attorney for Health and Welfare (LPA) or court order.

**Table 2:** Decision-maker selected.

	Vignette 1 n (%)	Vignette 2 n (%)	Vignette 3 n (%)	Vignette 4 n (%)	Vignette 5 n (%)
No one	14 (11%)	11 (9%)	7 (5%)	15 (12%)	6 (5%)
Person featured in the vignette (patient or care home resident)	49 (39%)	41 (32%)	30 (24%)	32 (25%)	46 (36%)
Family member described in vignette (daughter, son or mother)	46 (36%)	**43 (39%)**	57 (45%)	60 (47%)	**48 (38%)**
Medical professional described in the vignette (general practitioner/consultant)	17 (13%)	28 (22%)	26 (20%)	38 (30%)	33 (26%)
Multidisciplinary team following a best interests decision meeting	68 (53%)	75 (59%)	68 (53%)	44 (35%)	75 (59%)
The researcher	**14 (11%)**	10 (8%)	**13 (10%)**	**16 (13%)**	8 (6%)
I don’t know	4 (3%)	7 (5%)	11 (9%)	8 (6%)	7 (5%)
Other	2 (2%)	6 (5%)	3 (2%)	2 (2%)	3 (2%)
Care home manager*	–	–	25 (20%)	–	13 (10%)

Participants selected response for legally authorised decision-maker, shown by scenario. Participants were able to select more than one response in all vignettes. Bold values indicate the option that was congruent with the legal frameworks in that vignette.

*Option in vignettes 3 and 5 only.

### Concordance with the legal framework

Very few responses were wholly congruent with the legal frameworks. Most participants provided a mixture of responses across all five vignettes, including discordant, concordant, uncertain and mixed or unclear responses (table 3).

**Table 3:** Type of response by scenario.

	Vignette 1 n (%)	Vignette 2 n (%)	Vignette 3 n (%)	Vignette 4 n (%)	Vignette 5 n (%)
Discord	104 (82%)	96 (76%)	96 (76%)	104 (82%)	94 (74%)
Concord	12 (9%)	15 (12%)	10 (8%)	10 (8%)	23 (18%)
Wholly	5	6	5	1	5
Partially	7	9	5	9	18
Uncertain	5 (4%)	7 (5%)	12 (9%)	7 (5%)	4 (3%)
Unclear/mixed	6 (5%)	9 (7%)	9 (7%)	6 (5%)	7 (5%)

Responses categorised as demonstrating discord or concord with the legal frameworks, or where the participant was uncertain or response was unclear, shown by scenario.

Half of the participants (46%, 58/127) provided responses that were discordant with the legal frameworks in all five scenarios. Only two participants (2%) provided concordant responses across all five scenarios, both were from a nursing background and actively involved in research which included recruiting participants for studies. Twenty-four participants provided ‘I don’t know’ or uncertain responses to one or more scenarios, including one participant who responded that they were uncertain in all five vignettes. Vignette 3, which concerned a low-risk study of a communication device being trialled in a nursing home, had the highest number of uncertain responses (9%, 12/127).

Analysis of the factors cited as relevant to the scenario revealed that the family member was selected as the decision-maker as they were considered to be the ‘next of kin’. Where this was cited, it was more commonly in responses by participants from a healthcare professional background (93%, 27/29) compared with those from social care practitioners (7%, 2/29). Selection of the multidisciplinary team (MDT) as decision-maker following a best interests meeting was common and was the predominant option in all the scenarios apart from vignette 4 which involved a study requiring access to medical notes and investigation results. Responses indicated that participants were unclear whether consent was required (or not) prior to accessing medical notes for research purposes.

‘Best interests’ was commonly reported as a factor relevant to the scenario in the open-text responses (n=135) by both responses from healthcare professionals (67%, 90/135) and social care practitioners (33%, 45/135). One participant (a senior social care practitioner) was concerned that the proposed intervention in vignette 3 may be viewed as restrictive of the man’s liberty and questioned whether Deprivation of Liberty safeguards were in place. The same participant felt that the appropriate decision-maker was the nurse assessor from the National Health Service body that would commission his care in the nursing home. Another participant (a senior social care practitioner) stated that, as there was no person empowered to make the decision, the matter may need to be brought before a judge.

### Relevant variables in decision-making

Neither the level of perceived risk nor the study type appeared to affect the level of knowledge or understanding. When comparing professional groups, nurses consistently had the highest proportion of responses concordant with the legal frameworks, between 30% and 50% depending on scenario, although they were also the largest professional group. Social care practitioners provided the highest proportion of responses that were discordant in all scenarios (71%, 20/28), although there were much lower levels of involvement in research (36%, 10/28) compared with other professional groups. GPs were commonly cited as the authorised decision-maker by other groups, as well as GPs themselves, with the exception of a postoperative medication trial in a hospital setting (vignette 2). However, GPs’ own responses were no more concordant than other groups, with 54% (7/13) providing responses that were discordant across all five scenarios, and others expressing degrees of uncertainty. Prior involvement in research did not appear to affect the level of knowledge or understanding. Health and social care professionals who led research studies as either a chief investigator of a study or a principal investigator at a site did not provide responses that were more concordant than those who did not have a responsibility for leading studies; with 40% (10/25) providing discordant responses across all scenarios.

## Discussion

The levels of knowledge of the legislation governing research involving adults lacking capacity found in this study were predominantly low, which raises concerns about the accessibility of research and opportunity to participate for those who lack capacity, the ability to conduct research involving such groups, and the impact on the evidence base for their care.

### Legally authorised decision-maker

The findings demonstrated low levels of knowledge of the dual legislation governing research involving adults lacking capacity, which supports previous concerns about the complexity of the regulatory framework.^4 21^ Participants generally did not distinguish between research involving medicinal products and other types of research, nor between the processes of consultation and consent. Participants did not recognise that the researcher is the decision-maker under MCA,^2^ or that the MCA provisions enable recruitment *without consent* as there are additional legal safeguards in place such as stringent ethical review. For the two vignettes that involved a clinical trial of a medicinal product (vignettes 2 and 5), more participants identified the close family member as the decision-maker,^3^ although not necessarily recognising that they were providing legally valid informed consent on the person’s behalf.

Of concern was the lack of understanding that a person who knew the person well in a personal capacity was legally authorised to be the decision-maker under CTR. Or that an LPA or court order was not required. This response was more frequently provided by social care practitioners, rather than health or allied healthcare professionals, and was reported by those considered to be senior practitioners (>8 years in their role). An LPA for Health and Welfare is generally limited to health and care decisions, rather than medical research; moreover, an LPA may not be possible for those who have never had mental capacity for making such decisions. The individual acting as personal consultee or legal representative does not need to be a legally appointed attorney or deputy, the MCA merely states that

The fact that a person is the donee of a lasting power of attorney given by P, or is P’s deputy, *does not prevent* him from being the person consulted under this section. s32(7)^2^

The role of the attorney in providing consent for medical research has been reported elsewhere to be unclear.^22^ It may be considered good practice to consult the attorney as part of the requirement to seek the views of any carers and other relevant people before involving a person who lacks capacity in research (s7.57, s11.20).^20^ However the researcher (in accordance with the advice from the consultee) or the legal representative is the legally authorised decision-maker.

### Ethical basis for the decision

A second key finding was that participants widely reported that the basis for the decision, regardless of who made the decision, was what was considered to be in the person’s best interests. The legal basis for the decision is the ‘substituted judgement’ standard which requires proxies to make decisions that reflect the patient’s views and values.^23^ Best interests is the dominant standard for treatment and care decisions, but not for research according to the legal framework in England and Wales^20^ and beyond.^23^ It is considered an ethically weak basis for enrolling those without capacity into research which is not intended (or likely) to benefit them.^23^While it is likely that a person acting as a consultee or legal representative will be concerned for the welfare and interests of the person who they are acting on behalf of, this is not necessarily what is in their *best* interests. Although disentangling a substituted judgement from concern for the person’s interests may be problematic for proxies generally, and indeed their concern for the person’s interests is the reason they are involved in such decisions.

The source of this misunderstanding may be that the MCA is now firmly embedded in treatment and care decision-making for adults lacking capacity. This includes the fourth statutory principle which requires that an act done, or decision made, on behalf of a person who lacks capacity must be done, or made, in his best interests (s1(5)).^2^ This is qualified by the MCA Code of Practice as ‘the only exceptions to this are around research and advance decisions to refuse treatment where other safeguards apply’ (s2.12).^20^ However, the failure to emphasise the crucial difference between research decisions and all others, and clarify the ambiguity regarding the degree to which best interests is involved in research decisions, appears to have led to confusion and uncertainty about the role of ‘best interests’ in research involving adults who lack capacity.

### Comparison with existing research

The findings from this survey are consistent with studies in other jurisdictions. Uncertainty about how the legislation should be interpreted has also been reported in a survey of care home managers and key informant interviews exploring research in care homes.^24^ Bravo *et al* 25 surveyed Canadian researchers and found that there was a lack of awareness about who can act as proxy decision-maker for research.^25^ They called for greater clarity and education about who can act as proxy decision-maker.^25^ An earlier study led by the same author examined knowledge of the legislation governing proxy consent to both treatment and research of four Canadian groups (older adults, informal caregivers of cognitively impaired individuals, researchers in ageing and members of research ethics boards).^15^ They found that knowledge of proxy consent for research was lower (from 2% among older adults, 36% among researchers, to 44% among ethics board members) for the scenario describing research involving an adult lacking capacity who did not have a legal guardian. They recommended that more education, including public awareness campaigns, was needed.^15^ A US survey of clinical investigators into Alzheimer’s disease found that many of those surveyed either did not know or incorrectly thought no laws or regulations existed regarding who has the authority to provide proxy informed consent in their state.^16^ They also found that respondents who asserted that no one can provide proxy informed consent also reported that very high proportions of their patients were capable of providing adequate informed consent. They concluded that further training on regulations governing research involving adults lacking capacity may be needed, and interventions to improve informed consent which included all those involved.

### Limitations

Limitations of this study include that this was a self-completed online survey, which may have resulted in selection and response biases. Although participants were required to confirm their status as a health or social care professional, it could not be independently verified that participants responding via social media platforms held positions in health and social care. The high proportion of participants from Wales, compared with England, reflected the geographical location of the research team and some networks that disseminated the survey. The question wording ‘legally authorised’ may have been interpreted as a specific transfer of power or decision-making authority, rather than who is authorised according to the legal framework, although the phrase has been successfully used in a similar survey.^15^ Participants may have selected all those they thought should be included in the discussion, rather than the decision-maker as specified in the task.

Eligibility was not restricted to those already experienced in research as a range of views was sought, and there is an increasing expectation that research activity will become embedded across the whole health and social care system. Health and social care professionals may also be approached to act as a nominated consultee or professional legal representative for those in their care, if no family member or friend is willing or able to act as a personal consultee or legal representative. However, the inclusion of some health and social care professionals who had limited or no experience of research means they may not have understood the nature of research, conflating it with medical treatment which is intended to benefit the person directly.

### Conclusions

Research involving adults lacking capacity is complex; decision-making processes differ from usual care and treatment decisions, and between different types of research. Participants demonstrated a lack of knowledge about the locus of authority; viewing MDTs as the only authorised decision-makers, who were entitled to make decisions in accordance with the person’s best interests, as is the case for medical treatment and care. Family members and researchers were consigned to information provider roles, although some responses indicated that they included family members in the MDT.

The vast majority of participants did not recognise that the basis for enrolling an adult who lacks capacity in research is what the person themselves would have wanted if they had capacity to decide, their ‘presumed will’. Participants did not understand or acknowledge that the family member was best placed to advise, or decide, what the person’s wishes would have been. Whether participating was in the person’s ‘best interests’ was overwhelmingly cited as the ethical basis for the decision. This suggests that the standard has become ubiquitous in decision-making for adults lacking capacity, regardless of the legal validity in situations such as research participation decisions. This conflict arises when what is in the person’s best interests, as a form of beneficence, is given precedence over autonomy.

Appropriate mechanisms for involving adults lacking capacity in research are vital if such groups are to have an equal opportunity to participate as all other members of society. The low levels of health and social care professionals’ knowledge and understanding described in this study are a concern if legally and ethically legitimate enrolment processes are not adhered to. Health and social care professionals’ experiences and application of the legal frameworks in practice, and attitudes towards the inclusion of those with incapacity in research, may warrant further in-depth exploration. The findings from this survey suggest that there is a pressing need for interventions to improve levels of legal literacy, including enhanced education and training which focuses on the legal frameworks governing research involving adults who lack capacity to consent.
