# Supplementary material for: Healthcare professionals’ understanding of the legislation governing research involving adults lacking mental capacity in England and Wales: a national survey
Source: J Med Ethics. 2018 Apr 25;44(9):632–7. doi: 10.1136/medethics-2017-104722 (PMC6119350; doi:10.1136/medethics-2017-104722)
Supplement: Supplementary data [file medethics-2017-104722supp001.pdf]

APPENDIX 1 SURVEY VIGNETTES

Scenario 1

Mrs Jones has advanced dementia, and because of her condition she is unable to understand when something is explained to her. Since her husband died she has lived with her daughter. There is no Lasting Power of Attorney or court order in place. A researcher from the University is doing a study to look at whether classical music has a beneficial effect on people with dementia. The study does not involve any serious risk for the participants. The researcher would like Mrs Jones to take part.

Scenario 2

Mrs Jones (from scenario 1) is admitted to the local hospital with a fractured femur. An anaesthetist is carrying out a clinical trial into a new medication to relieve postoperative pain that may result in less side effects, such as confusion, in patients with dementia. The known side effects of the medication include nausea and headache and, rarely, kidney or liver failure. Mrs Jones is assessed by her consultant as eligible for the trial.

Scenario 3

Mr Smith is living in a nursing home following a severe stroke that has left him unable to understand information given to him, or communicate in any way. A company have developed a device that monitors facial expressions to help staff understand how a person may be feeling (pain etc) and are carrying out a research study which Mr Smith's care home is taking part in. The study does not involve any serious risk for the participants. His son now lives a long way away, but visits every two months and speaks to the care home staff weekly on the phone.

Scenario 4

Mr Smith (from scenario 3) is admitted from the care home to the Intensive Care Unit of the local hospital with pneumonia. The University is conducting a study looking at all pneumonia admissions to hospital from care homes in the area. The study involves accessing patients' medical notes and results of investigations. The study does not involve any serious risk for the participants. His son is staying nearby while his father is in hospital and visits daily.

Scenario 5

Miss Lewis is 21 years old and has profound and multiple learning disabilities and lives in a small residential care home close to her mother who visits most days. There is no Court of Protection order in place. She is on long term medication intended to control muscle contractures. This medication may not be effective for this type of problem and can result in side effects. Her General Practitioner is taking part in a study to look at whether such medication can be reduced or withdrawn safely without any worsening of symptoms. There may be some risks for the participant.
